# Editorial: Machine and deep-learning for computational neuroscience

**DOI:** 10.3389/fncom.2023.1218895

**Published:** 2023-07-04

**Authors:** Gaurav Dhiman, Wattana Viriyasitavat, Atulya K. Nagar

**Affiliations:** ^1^Department of Electrical and Computer Engineering, Lebanese American University, Byblos, Lebanon; ^2^University Centre for Research and Development, Department of Computer Science and Engineering, Chandigarh University, Mohali, India; ^3^Department of Computer Science and Engineering, Graphic Era Deemed to be University, Dehradun, India; ^4^Division of Research and Development, Lovely Professional University, Phagwara, India; ^5^Chitkara University Institute of Engineering and Technology, Chitkara University, Chandigarh, Punjab, India; ^6^Department of Computer Science, Government Bikram College of Commerce, Patiala, India; ^7^Faculty of Commerce and Accountancy, Chulalongkorn University, Bangkok, Thailand; ^8^Liverpool Hope University, Liverpool, United Kingdom

**Keywords:** machine learning, deep learning, neuroscience, classification, disease

The field of computational neuroscience has rapidly evolved over the past few decades, fueled by advancements in technology and the accumulation of large-scale neural data (Shen and Saab, 2021; Shen et al., 2021; Abdellatef et al., 2022; Sayour et al., 2022). With the emergence of machine and deep-learning techniques, there has been a shift toward utilizing these methods to extract insights from complex neural data, leading to unprecedented progress in understanding the brain's inner workings (Helwan et al., 2021; Saab and Jaafar, 2021; Saab et al., 2021; Hammoud et al., 2022). Machine and deep-learning approaches (Abbas et al., 2021; Gerges et al., 2021; Tarhini et al., 2022) offer several advantages over traditional statistical techniques, including their ability to handle large and complex datasets, learn from data, and make predictions based on patterns and relationships within the data. Machine and deep-learning techniques have been applied to various areas of computational neuroscience, including brain-computer interfaces, neuroimaging, and neural decoding (Tarhini et al., 2020; Hammoud et al., 2021; Sorkhoh et al., 2021). These approaches have led to new insights into brain function and the development of novel diagnostic and therapeutic tools (Chamra and Harmanani, 2020; Fakhoury et al., 2022) for neurological disorders. For example, deep-learning models have been used to accurately predict epileptic seizures, diagnose Alzheimer's disease, and analyze neural network dynamics (Prakash and Lina, 2021; Senay et al., 2021; Tohme and Martin, 2021; Tohme et al., 2021). In this Research Topic, we accepted six papers that showcase the latest advancements in machine and deep-learning for computational neuroscience.

The first paper, “Transfer learning-based modified inception model for the diagnosis of Alzheimer's disease (Sharma et al.),” proposes a new deep-learning model that utilizes transfer learning to diagnose Alzheimer's disease with high accuracy. The model is based on a modified version of the inception model and was trained on a large dataset of brain images. The results demonstrate that the proposed model outperforms traditional machine learning methods and can be a useful tool for early diagnosis of Alzheimer's disease.

The second paper, “A multi-frame network model for predicting seizures based on sEEG and iEEG data (Lu et al.),” presents a new deep-learning model that can predict seizures with high accuracy using intracranial and scalp electroencephalogram (EEG) data. The model is based on a novel multi-frame network architecture and was trained on a large dataset of epilepsy patients. The results demonstrate that the proposed model can accurately predict seizures up to 20 seconds in advance, which could be a game-changer for epilepsy treatment.

The third paper, “Analysis of instantaneous brain interactions contribution to a motor imagery classification task (Cristancho Cuervo et al.),” investigates the contribution of instantaneous brain interactions to a motor imagery classification task. The study uses a machine learning approach to analyze the interactions between brain regions during a motor imagery task. The results demonstrate that instantaneous brain interactions play a crucial role in motor imagery classification and could be used to improve brain-computer interfaces.

The fourth paper, “Research on the network handoff strategy based on the best access point name decision (Shu et al.),” proposes a new machine learning-based network handoff strategy for wireless communication networks. The model uses a decision tree algorithm to select the best access point for a mobile device, which could improve the quality of service for wireless users.

The fifth paper, “Improved space breakdown method—A robust clustering technique for spike sorting (Ardelean et al.),” presents a new clustering technique for spike sorting. The method uses a machine learning approach to improve the accuracy of spike sorting, which is a crucial step in analyzing neural data. The results demonstrate that the proposed method outperforms traditional spike sorting methods and could be a useful tool for studying neural circuits.

The sixth and final paper, “Stability of mental motor-imagery classification in EEG depends on the choice of classifier model and experiment design, but not on signal preprocessing (Rosenfelder et al.),” investigates the stability of mental motor-imagery classification in EEG. The study compares different machine learning classifiers and experimental designs and finds that the choice of classifier and experimental design have a significant impact on the stability of the classification, while signal preprocessing does not.

In summary, the papers presented in this Research Topic demonstrate the power of machine and deep-learning for computational neuroscience. These approaches have the potential to revolutionize our understanding of brain function and improve diagnosis and treatment of neurological disorders. We hope that this Research Topic will inspire further research in this exciting and rapidly evolving field.

## Author contributions

All authors have equally contributed in this Research Topic. All authors contributed to the article and approved the submitted version.
